# The value of glucocorticoid co-therapy in different rheumatic diseases - positive and adverse effects

**DOI:** 10.1186/ar4686

**Published:** 2014-11-13

**Authors:** Marlies C van der Goes, Johannes W Jacobs, Johannes W Bijlsma

**Affiliations:** 1 Department of Rheumatology & Clinical Immunology (F02.127), University Medical Center Utrecht, PO Box 85500, 3508 GA Utrecht, the Netherlands

## Abstract

Glucocorticoids play a pivotal role in the management of many inflammatory rheumatic diseases. The therapeutic effects range from pain relief in arthritides, to disease-modifying effects in early rheumatoid arthritis, and to strong immunosuppressive actions in vasculitides and systemic lupus erythematosus. There are multiple indications that adverse effects are more frequent with the longer use of glucocorticoids and use of higher dosages, but high-quality data on the occurrence of adverse effects are scarce especially for dosages above 10 mg prednisone daily. The underlying rheumatic disease, disease activity, risk factors and individual responsiveness of the patient should guide treatment decisions. Monitoring for adverse effects should also be tailored to the patient. Continuously balancing the benefits and risks of glucocorticoid therapy is recommended. There is an ongoing quest for new drugs with glucocorticoid actions without the potential to cause harmful effects, such as selective glucocorticoid receptor agonists, but the application of a new compound in clinical practice will probably not occur within the next few years. In the meantime, basic research on glucocorticoid effects and detailed reports on therapeutic efficacy and occurrence of adverse effects will be valuable in weighing benefits and risks in clinical practice.

## Introduction

The value of glucocorticoid therapy was discovered 65 years ago. In 1949, Philip Hench reported the dramatic effect of compound E on rheumatoid arthritis (RA) [1]. Nowadays, glucocorticoids play a pivotal role in the management of many inflammatory rheumatic diseases [2-7]. Glucocorticoids represent the most frequently employed class of anti-inflammatory drugs, with a rise in use in recent years [8-11], despite the development of new treatment modalities such as biological drugs applicable for many rheumatic diseases. Community survey data indicate use of glucocorticoids among 0.5% of the general population, and 1.4% of women aged older than 55 years [12,13]. Between 14.6 and 90% of patients with RA worldwide are undergoing treatment with glucocorticoids [10]. Almost all patients with polymyalgia rheumatic, giant cell arteritis and systemic vasculitis use systemic glucocorticoids.

**Figure 1:**
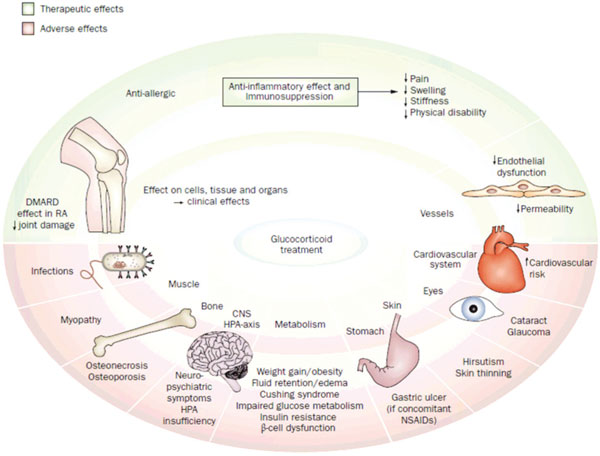
**Effects of glucocorticoids**. Glucocorticoid therapy is associated with both beneficial effects (upper part) and adverse effects (lower part). CNS, central nervous system; DMARD, disease-modifying antirheumatic drug; HPA, hypothalamic-pituitary-adrenal; NSAID, nonsteroidal anti-inflammatory drug; RA, rheumatoid arthritis. Adapted from [127].

Glucocorticoids are very effective anti-inflammatory and immunosuppressive drugs, but their use is restrained by fear for and occurrence of adverse effects (Figure 1).

## Mechanisms of action

**Figure 2:**
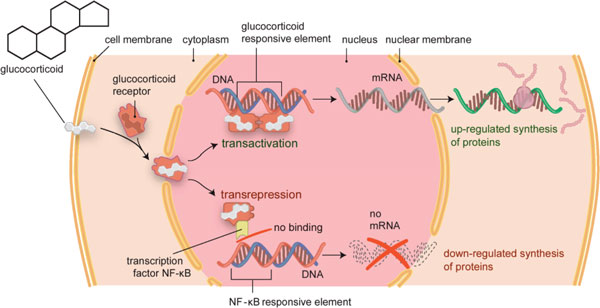
**Genomic action of glucocorticoids**. Glucocorticoid binds to the glucocorticoid receptor (GCR) in the cytoplasm. This complex migrates into the nucleus. Activation of transcription (transactivation) by binding of GCR-glucocorticoid complex dimers to glucocorticoid-responsive elements of DNA upregulates synthesis of regulatory proteins, thought to be responsible for metabolic effects and also some anti-inflammatory/ immunosuppressive effects. Interaction of GCR-glucocorticoid complex monomers with proinflammatory transcription factors, such as activator protein-1, interferon regulatory factor-3 and nuclear factor (NF)-κB, leads to inhibition of binding of these transcriptional factors to their DNA consensus sites (for NF-κB: NF-κB-responsive elements). The transcription of these proinflammatory transcription factors is thus repressed. This process is called transrepression and downregulates synthesis of predominantly inflammatory/immunosuppressive proteins. Adapted from [128].

The effects of glucocorticoids are thought to be mediated by different mechanisms [14-19]. Classic genomic mechanisms leading to changes in gene expression are most important in low-dose therapy (see Figure 2) [20]. These actions occur after passage of glucocorticoid molecules through the cell membrane, binding to the inactive cytosolic glucocorticoid receptor, formation of an activated glucocorticoid-cytosolic glucocorticoid receptor complex, and translocation of this complex into the nucleus. Transactivation and transrepression can be initiated in the nucleus. Transactivation is caused by binding of two activated glucocorticoid-cytosolic glucocorticoid receptor complexes as a dimer to the promotor of glucocorticoid-regulated genes, which leads to up-regulation of the synthesis of certain regulatory proteins by transcriptional activation. In transrepression, glucocorticoid-cytosolic glucocorticoid receptor monomer complexes interfere with the activity of (proinflammatory) transcription factors such as activator protein 1 and nuclear factor-κB, leading to downregulation of (proinflammatory) protein synthesis. Genomic processes require at least 30 minutes before changes can be observed in regulatory protein synthesis, but usually it will take hours to days for changes to occur at the cell, tissue or organ level [18,21].

The repressive, anti-inflammatory effects of glucocorticoids were believed until recently to be based mainly on transrepression [19], whereas the negative effects - with the exception of infection risk - and metabolic actions of glucocorticoid therapy were based on transactivation. This view has been somewhat revised, with the suggestion that some key anti-inflammatory actions of glucocorticoids are caused by gene activation [19,22]. Moreover, research in a mouse strain with a deficiency in forming dimer glucocorticoid-glucocorticoid receptor complexes, and thus with a transactivation deficiency, showed - along with a failure of glucocorticoids to exert a full anti-inflammatory response - classic side effects such as osteoporosis in these mice [23].

In very high-dose therapy, nongenomic mechanisms also occur, effects of which are evident within minutes [20]. These nongenomic mechanisms are mediated via the cytosolic glucocorticoid receptor, via the membrane-bound glucocorticoid receptor, and via nonspecific interactions with membranes of cells and organelles, including those of mitochondriae.

The size of genomic effects of glucocorticoid preparations defines their dose equivalence. Dexamethasone and betamethasone are the most potent preparations, followed by methylprednisolone, predniso(lo)ne, cortisol and cortisone [20]. Dosages of specific glucocorticoid preparations often are expressed in relation to the potency of the most frequently used preparation; that is, prednisone. The definitions of low-dose therapy through to pulse therapy are presented in Table 1.

**Table 1:** Definition of conventional terms for glucocorticoid dosages.

Dose	Definition
Low	≤7.5 mg prednisone equivalent/day
Medium	>7.5 mg but ≤30 mg prednisone equivalent/day
High	>30 mg but ≤100 mg prednisone equivalent/day
Very high	>100 mg prednisone equivalent/day
Pulse therapy	≥250 mg prednisone equivalent/day for 1 day or a few days

Adapted from [20].

## Patterns of glucocorticoid use

Glucocorticoid use (dose, duration, administration) depends on the diagnosis, indications for glucocorticoid therapy, and the goal of treatment (see Table 2). The therapeutic effects range from pain relief in arthritides, to disease-modifying effects in early RA, and to strong immunosuppressive actions in vasculitides and systemic lupus erythematosus (SLE). Not all effects have been studied in detail.

**Table 2:** General use of glucocorticoids in rheumatology.

	Initial oral dose			Intravenous, very high dose or pulse therapy	Intra-articular injection
	**Low**	**Medium**	**High**		
			
Arthritides					
Gouty arthritis, acute	-	2	2	-	2
Juvenile idiopathic arthritis	-	1	1	-	1
Osteoarthritis	-	-	-	-	1
Acute calcium pyrophosphate crystal arthritis	-	-	-	-	2
Psoriatic arthritis	-	1	-	-	2
Reactive arthritis	-	-	-	-	1
Rheumatic fever	-	1	1	-	-
Rheumatoid arthritis	2	2	1	1	2
Collagen disorders					
Dermatomyositis, polymyositis	-	-	3	1	-
Mixed connective tissue disease	-	1	-	1	1
Polymyalgia rheumatica	-	3	-	1	-
Sjögren's syndrome, primary	-	-	1	-	-
Systemic lupus erythematosus	-	2	1	1	-
Systemic sclerosis	-	1	-	-	-
Systemic vasculitides					
In general	-	-	3	1	-

Initial dose, the dose at the start of therapy, will often be decreased in time depending on disease activity: low, ≤7.5 mg prednisone equivalent/day; medium, >7.5 but≤30 mg prednisone equivalent/day; high, >30 but ≤100 mg prednisone equivalent/day; very high, >100 mg prednisone equivalent/day. -, rare use; 1, infrequent use, for therapy-resistant disease, complications, severe flare, major exacerbation, and for bridging the lag-time of recently started therapy; 2, frequently added to/used as the basic therapeutic strategy; 3, basic part of the therapeutic strategy. With permission from BMJ publishing group [129].

### Primary immunosuppressive treatment with glucocorticoids

Glucocorticoids are anchor drugs for therapeutic strategies in myositis, polymyalgia rheumatica and systemic vasculitis. For other rheumatic diseases, glucocorticoids are adjunctive therapy, or are not used at all. Mono-therapy with glucocorticoids is applied in polymyalgia rheumatica; remission can often be achieved with treatment starting at 15 mg prednisone or equivalent daily [24].

Glucocorticoids also represent the most important class of drugs in giant cell arteritis. For this disease, higher initial dosages are often used (mostly 40 to 60 mg prednisone or equivalent daily). Furthermore, in the case of (transient) acute visual loss, pulse therapy is applied.

In the treatment of polymyalgia rheumatica and giant cell arteritis, other immunomodulatory drugs are sometimes added to glucocorticoids as glucocorticoid-sparing agents to decrease the dose or duration of glucocorticoid therapy. However, the results of research into the glucocorticoid-sparing properties of other immunosuppressive medication - that is, use of other agents to decrease the cumulative glucocorticoid dose - are not convincing. Outcomes of randomized controlled trials in polymyalgia rheumatica on the glucocorticoid-sparing effects of methotrexate [25-27], azathioprine [28] and infliximab [29] were conflicting. The same holds true for adjunctive treatment with methotrexate [30-32], cyclosporine [33], etanercept [34] and infliximab [35] in giant cell arteritis. A recent retrospective study in 23 patients indicated that leflunomide is effective as a glucocorticoid-sparing agent in patients with difficult-to-treat giant cell arteritis and polymyalgia rheumatic, but this still needs to be confirmed in randomized controlled trials [36].

### Combination therapy including glucocorticoids

#### Pulse therapy

Acute episodes or particularly severe forms of rheumatic diseases and specific complications are indications for pulse therapy. In collagen disorders, pulse therapy is applied for disease induction or treatment of flares. In RA, pulse therapy is used to treat serious complications of the disease and to induce remission in active disease. In the latter, it makes sense to simultaneously start a new second-line antirheumatic treatment aiming at stabilizing the remission induced by pulse therapy, since the beneficial effects generally last about 6 weeks [37]. There are several different schemes of pulse therapy. Pulse therapy with schemes of 1,000 mg methylprednisolone intravenously has proven effective in many studies.

#### High and medium doses

In systemic vasculitis, high-dose glucocorticoids are the cornerstone of induction therapy and the therapy of flares. Usually, other immunosuppressive drugs are applied simultaneously. In this chronic treatment, the dosages (often starting with 1 mg/kg body weight prednisone equivalent) are gradually reduced in weeks to months to a maintenance therapy of low to medium dose.

Another application of high-dose glucocorticoids is intermittent treatment; for example, in gout to treat attacks. In a gout attack, treatment with 35 mg prednisone during 5 days improved pain scores, as did treatment with naproxen [38]. Glucocorticoids are stopped in the periods between these attacks, although there are a few exceptions. For example, glucocorticoids can be used in patients with gout as a bridge for adequate suppression of uric acid in cases where nonsteroidal anti-inflammatory drugs (NSAIDs) and colchicine are contraindicated.

#### Low dose

Chronic glucocorticoid therapy in RA is often started and maintained at a low dose, mostly in addition to other disease-modifying antirheumatic drugs. Glucocorticoid therapy is highly effective for relieving symptoms in patients with active RA in the short and medium term [39,40]. Improvement has been documented for all clinical parameters, including pain scales, joint scores, morning stiffness and fatigue, but also in parameters of the acute phase reaction (that is, erythrocyte sedimentation rate and C-reactive protein). Use of a modified-release preparation at bedtime (that is, releasing the administered glucocorticoid in the early morning, similar to the circadian rhythms of endogenous cortisol) leads to even more improvement in morning stiffness than use of a conventional tablet of equal dosage taken after awakening [41]. After 6 months of therapy, the beneficial effects of glucocorticoids in general seem to diminish. However, clinical experience suggests that if this therapy is then tapered off and stopped, some patients experience clear aggravation of symptoms and signs, indicating that the beneficial effect was still present before tapering. In reports, very low doses of glucocorticoids (that is, <5 mg prednisone equivalent/day) can be sufficient for patients to remain in remission without causing severe adverse events [42]. However, the risk-benefit ratio of this therapy with very low doses has not been studied in a prospective randomized way.

Recent studies have (re)established the disease-modifying potential of low-dose glucocorticoids in early RA and have renewed the debate on benefits and risks of this treatment [43-52]. Beneficial effects of initial glucocorticoid treatment during the first 2 years of the disease on retardation of joint damage are still present after 4 to 5 years [53-55], which has not been shown for any other disease-modifying antirheumatic drug. As such, glucocorticoids can be used for helping to achieve remission and prevent joint damage in the long term; they are part of the management for early RA according to European guidelines [56]. Surprisingly, evidence for the efficacy of glucocorticoids was not incorporated in the 2012 update of the American College of Rheumatology treatment recommendations for RA [57], which is highly debatable [58].

### Local application of glucocorticoids

Another type of use is local application of glucocorticoids. In persisting nonbacterial arthritis of a joint, intra-articular glucocorticoid injection can be considered. The effect depends on several factors, such as the treated joint (size, weight bearing, or non-weight bearing), the activity of arthritis and the volume of synovial fluid in the treated joint, the application of arthrocentesis (synovial fluid aspiration) before injection, the choice and dose of the glucocorticoid preparation, the injection technique, and application of rest to the injected joint [59].

Among the injectable glucocorticoids, triamcinolone hexacetonide is the least soluble preparation and showed the longest effect [60]. In a randomized controlled study, bed rest for 24 hours following injection of a knee joint in patients with inflammatory arthritis resulted in pro-longed duration of clinical response and reduced the need for additional injections, compared with a control group that received no particular advice about activity with the injected joint [61]. Favorable effects of resting the injected joints (for example, by splinting in a cast or plaster) for 3 weeks in the case of a non-weight bearing (upper extremity) joint and 6 weeks for a weight bearing (lower extremity) joint have been described [62]. Based on the literature, no definite evidence-based recommendation can be made, but it seems prudent to rest and certainly not to overuse the injected joint for several days, even if pain is relieved [63].

An alternative to glucocorticoid therapy for intra-articular use has not yet been recognized.

## Adverse effects

There is no doubt that glucocorticoids have a high potential for frequent and serious adverse events. Compared with other antirheumatic agents, glucocorticoids have a low incidence of short-term symptomatic toxicity and patients uncommonly discontinue therapy for this reason. There are multiple indications that adverse effects are more frequent with longer use of glucocorticoids and use of higher dosages [64,65], but high-quality data on the occurrence of adverse effects are scarce especially for dosages above 10 mg prednisone daily. Most published studies on glucocorticoid toxicity are retrospective and observational [66]. Systematic reviews and randomized controlled trials are considered the highest quality evidence, but these studies are often focused on treatment efficacy. They have not been powered or designed to assess toxicity. The inability to differentiate unfavorable outcomes attributable to glucocorticoids from those occurring as a complication of the disease treated or as co-morbidities also confounds the picture. Furthermore, there is strong selection bias for glucocorticoid use because physicians are inclined to treat patients with more severe disease with (higher dosages of) glucocorticoids (confounding by indication).

Therefore, despite over 60 years of use, robust data on toxicities of long-term glucocorticoid therapy are sorely lacking [67-70]. Fortunately, many of the adverse events can be avoided or dealt with if glucocorticoids are used prudently. An overview of positive as well as negative effects of glucocorticoid therapy is given in Figure 1.

In the following paragraphs, several notorious adverse effects of glucocorticoid therapy are discussed. These paragraphs will give an overview of frequently reported adverse effects in literature. Additionally, we will discuss the influence of disease and treatment scheme on the occurrence of adverse effects.

### Effects on bone

#### Osteoporosis

Chronic oral treatment with more than 5 mg prednisone daily can lead to a reduction in bone mineral density and an increase in the risk of fracture [71]. However, in many studies concerning glucocorticoid-induced osteoporosis, no attention is paid to the fact that glucocorticoids are usually prescribed for inflammatory diseases, which themselves have negative impact on bone mineral density.

**Figure 3:**
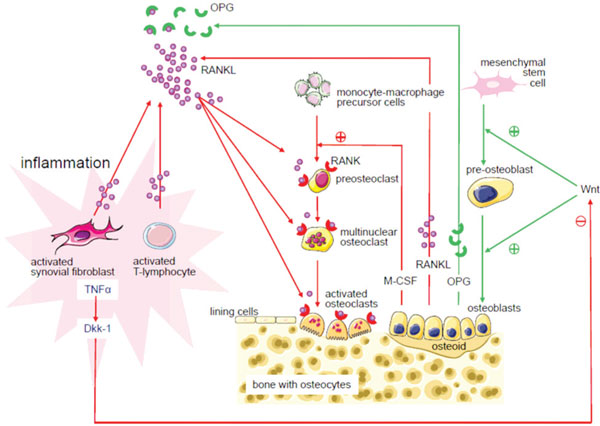
**Effects of inflammation on bone**. Processes stimulating bone growth (green arrows) and processes leading to bone loss (red arrows). The proinflammatory cytokine tumor necrosis factor alpha (TNFα) induces expression of Dickkopf-1 (Dkk-1) in synovial fibroblasts, which inhibits the Wnt signaling pathway. This results in decreased osteoblastogenesis and bone formation. Simultaneously, osteoclastic bone resorption is stimulated via receptor activator of nuclear factor-κB ligand (RANKL). Proinflammatory mediators stimulate osteoblasts to release macrophage colony-stimulating factor (M-CSF), stimulating osteoclastogenesis. Osteoprotegerin (OPG) is also released by osteoblasts and binds RANKL, thereby inhibiting osteoclastogenesis. RANK, receptor activator of nuclear factor-κB. Figure reproduced with permission from [130].

Bone mineral density changes may develop in RA in the absence of glucocorticoid therapy. Previous findings suggest that bone loss in RA patients not on glucocorticoid therapy mainly occurs during the first months of disease [72,73]. Correlations with parameters of inflammation have been found in some studies [74-77]. Development of osteoporosis can thus also be caused by active early RA. In early RA, glucocorticoids have positive effects on bone [43,44,46-51]. The joint-sparing effect is probably based on the inhibition of proinflammatory cytokines such as interleukin-1 and tumor necrosis factor [78,79], which have direct positive effects on osteoclast formation and stimulate osteoblasts and T lymphocytes to produce receptor activator of nuclear factor-κB ligand [78,80]. Binding of receptor activator of nuclear factor-κB ligand to its receptor (receptor activator of nuclear factor-κB) on osteoclast precursor cells leads to differentiation and activation of osteoclasts, and subsequently to bone resorption, periarticular osteopenia, and formation of bone erosions in patients with RA (see Figure 3) [78]. A recent study showed that the bone mineral density scores in the first years of treatment with low-dose glucocorticoids are similar to these scores in patients without glucocorticoid therapy, when preventive medication for osteoporosis has been used [77]. Preventive measures will be discussed in the section 'Recommendations for clinical practice'.

#### Osteonecrosis

For adverse effects on bone other than osteoporosis, a major limitation is that trials have not been powered or designed to assess toxicity or long-term effects, and therefore uncertainty of the true incidence and relevance of these adverse effects still remains. In general, osteonecrosis does not occur frequently. The frequency of occurrence clearly depends on the dose of glucocorticoid therapy and the underlying disease. Regarding pulse therapy, osteonecrosis seems to be a more frequent side effect in patients with SLE compared with patients with RA [81]. Both the disease and glucocorticoids therefore seem to play a role in osteonecrosis (especially) in SLE.

### Endocrine effects

#### Weight gain and moon face

In the case of long-term endogenous or exogenous glucocorticoid excess, patients can experience increased appetite. As a consequence, this may contribute to increased body and trunk fat, finally leading to a cushingoid appearance with centripetal fat accumulation and thin extremities with atrophic skin and contusions. A recent trial in RA with 10 mg prednisone daily showed a small but significant gain in weight over 2 years, with a mean of 2.9 kg in the methotrexate-based strategy group treated with prednisone compared with 1.3 kg in the methotrexate-based strategy group treated with placebo-prednisone [52]. Additional analyses showed that this body weight gain after treatment of active RA was, at least partly, regaining patients' previous weight by the reduction of the weight loss-inducing disease activity, which was more reduced in the prednisone strategy group [82].

The incidence of iatrogenic Cushing's syndrome is dependent on dose and duration of therapy. Development of a moon face is uncommon in therapy with sub-physiologic dosages.

#### Diabetes and cardiovascular disease

Many doctors and patients are afraid of the development of diabetes during glucocorticoid therapy [83], and of the thereby increasing risk of cardiovascular events [84]. Cardiovascular disease is more prevalent in rheumatic patients than in the general population [85]. This increased prevalence is probably based on elevated levels of proinflammatory cytokines such as tumor necrosis factor alpha and interleukin-6 in the systemic circulation, with multiple effects on organs, including adipose tissue, and endothelium [86]. These cytokines are also over-produced in dysfunctional adipose tissue of obese individuals [87]. The resulting cascade of changes throughout the body driven by systemic inflammation leads to a pro-atherogenic profile: atherogenic lipid abnormalities, oxidative stress, depletion of endothelial progenitor cells associated with impairment of vascular injury repair, increased arterial stiffness, insulin resistance, endothelial dysfunction, a hypercoagulable state, elevated homocysteine levels, and upregulation of atherogenic T cells [86].

In RA and SLE, markers of inflammation correlate with impaired insulin sensitivity and impaired beta-cell function [88-90]. Deterioration of glucose tolerance has not been observed for short-term glucocorticoid therapy at medium to high doses [91], which might be explained by the effectiveness of glucocorticoids in dampening the inflammation. The influence of glucocorticoid therapy at low to medium doses for multiple years on glucose metabolism seemed to be minor [92]. Based on these studies, one could conclude that glucocorticoids, if used to effectively treat an inflammatory condition, are not an important risk factor for the development of cardiovascular disease. However, in cases of overt diabetes before treatment, patients should be instructed to carefully monitor their blood glucose level shortly after starting glucocorticord therapy [69].

#### Suppression of the hypothalamic-pituitary-adrenal axis

Administration of glucocorticoids leads to negative feedback on the hypothalamus and pituitary glands, resulting in less secretion of corticotropin-releasing hormone and adrenocorticotropic hormone, respectively. As a result, the cortisol secretory capacity of the fasciculate-reticularis zone of the adrenal cortex may decrease, since this inner cortical zone of cortisol production is dependent on adrenocorticotropic hormone for structure and function.

Prediction of the suppression of the hypothalamic-pituitary-adrenal axis and tertiary adrenal insufficiency is not reliable, but their prevalence appears to depend on both the dose and duration of glucocorticoid treatment. In the case of long-term glucocorticoid therapy of up to 10 mg prednisone or equivalent daily, the risk of symptomatic adrenal failure is not high, but also not negligible. In clinical practice, it seems appropriate to anticipate potential adrenal insufficiency in patients who received 7.5 mg prednisone or equivalent for at least 3 weeks. Acute cessation of therapy could lead to problems. Gradual tapering of the glucocorticoid dose is therefore usually applied. In the case of suspected adrenal insufficiency, adrenocorticotropic hormone stimulation testing can help in evaluating the adrenal response [93]. Further, in the case of acute injury or stress (for example, with surgery), adequate adaptation of the glucocorticoid dose is important. Often a temporary increase of the dose to 15 mg prednisone equivalent/day is sufficient for minor surgery.

### Infections

Randomized clinical trials with glucocorticoids often report a not increased risk of infection [44,46,52,94,95]. However, cohort studies and case-control studies showed an increased occurrence of infections in patients with RA [96-99]. Moreover, the dose of glucocorticoid therapy and combination treatment with other immuno-suppressive drugs further elevate the risk [96]. Data suggest that awareness of the risk of infections before and during glucocorticoid treatment is needed.

Both typical and atypical microorganisms can cause infections. Clinicians should realize that glucocorticoids may blunt classical clinical features of infection, and thereby delay the recognition of the infection and the start of treatment. Opportunistic infections with *Pneumocystis jiroveci *have been reported for dosages from 16 mg prednisone equivalent daily during 8 weeks [100].

The performance of screening tests for infections, such as the tuberculin skin test and interferon gamma release assays, are suppressed by glucocorticoid therapy [101]. Furthermore, specific recommendations for vaccination in patients with inflammatory rheumatic diseases have been developed [102].

### Gastrointestinal problems

#### Bleeds

The risk of upper gastrointestinal tract bleeding or perforation increases about twofold with use of oral glucocorticoids or low-dose aspirin, and increases around fourfold with use of other NSAIDs [103]. This relatively high risk indicates that combined use of NSAIDs and glucocorticoids should be avoided, if possible [104]. If this combined treatment cannot be avoided, appropriate gastroprotective medication, such as proton pump inhibitors, helps to decrease the risk of bleeding and should be prescribed. In patients with low cardiovascular risk, cyclooxygenase-2 inhibitors may be an alternative for NSAIDs and proton pump inhibitors, since they induce a lower risk of gastrointestinal complications in RA compared with NSAIDs [105-107].

#### Other

Cases of bowel perforation and pancreatitis have been reported in the context of glucocorticoid use. However, causality between the glucocorticoid therapy and these events is unsure; other factors such as the inflammatory disease might also play a role.

### Neuropsychiatric events

Slight increases in sense of well-being are frequently reported by patients starting glucocorticoid therapy. Symptoms of acathisia, insomnia and depression have also been recorded. Similar to the risk of osteonecrosis, the risk of psychosis seems to be a more frequent side effect of pulse therapy in SLE patients compared with patients with RA [81]. Whether this increased incidence reflects an increased risk of this adverse effect of glucocorticoids in SLE or whether - and to what extent - this increased incidence also reflects psychosis as a complication of the disease is uncertain.

### Dermatologic problems

Easy bruising, ecchymoses, skin atrophy, striae, disturbed wound healing, acne and increased hair growth (excluding scalp hair) are undesirable dermatologic effects that occur with glucocorticoid therapy [108]. The physician often considers these changes to be of minor clinical importance, but they may be disturbing to the patient. No reliable data on the occurrence of these adverse effects are available.

### Ophthalmologic effects

Posterior subcapsular cataract is a well-described complication of prolonged glucocorticoid use [109]. This type of cataract is also related to diabetes, but it is not typically age related. Cortical cataracts also have also been associated with glucocorticoid use.

Glucocorticoid-treated patients may develop mild increased intra-ocular pressure, which can lead to minor visual disturbances. The development of frank glaucoma, threatening eye sight, is rare for low-dose therapy, and tends to appear in patients who are otherwise pre-disposed to the condition. A higher risk for glaucoma with glucocorticoids tends to occur more frequently if other risk factors are present, such as a family history of glaucoma, high myopia and diabetes [69]. In these patients, even when initiating low-dose therapy, an ophthalmologist should be consulted before the start of therapy [69].

## Recommendations for clinical practice

Safe glucocorticoid therapy is glucocorticoid treatment with optimal therapeutic effects and with minimal adverse effects. To facilitate safe use, recommendations for the management of glucocorticoid therapy in rheumatic diseases have been developed and have been published in recent years [68-70]. Adverse effects of glucocorticoids are partially avoidable. To avoid adverse events as much as possible, several measures can be taken.

### Education

First, patients should be informed of what to expect from this therapy. Nowadays, patients are more articulate and stand up for themselves. They should be actively informed about the expectations on both positive and negative effects of treatment with glucocorticoids. This information should be given over time, in small steps. Ideally, this should be supported by written information. Hopefully, this will lead to neutralizing of unfounded fears, earlier recognition of true adverse effects, and patient compliance.

### Preventive measures

Patients with or at risk of glucocorticoid-induced osteoporosis should receive appropriate preventive and/ or therapeutic interventions. In general, all patients starting glucocorticoid therapy at medium to high dose are at risk of developing osteoporosis. Calcium, (active) vitamin D and bisphosphonates have been proven effective in preventing and treating glucocorticoid-induced osteoporosis [110-115]. Preventive therapy with calcium and vitamin D should be started, because glucocorticoids impair bone mechanism - amongst other mechanisms - via inhibition of intestinal calcium absorption and renal tubular calcium reabsorption. Additionally, in general, bisphosphonates are indicated. Guidelines on indications and choices for specific drugs differ somewhat between countries.

Several algorithms have been developed to refine the estimate of the risk of fractures for individual patients, such as the Fracture Risk in Glucocorticoid-induced Osteoporosis score, which includes the glucocorticoid dosage taken, and FRAX^® ^fracture risk assessment [116-119], for which adjustments have also been suggested for glucocorticoid dosages >7.5 mg prednisone equivalent daily [120,121]. The decision of whether a patient should be treated depends on fracture risks and on the cost, effectiveness and safety of the treatment.

### Optimizing therapy

The initial dose, dose reduction and long-term dosing depend on the underlying rheumatic disease, disease activity, risk factors and individual responsiveness of the patient [68]. Except for treatment with glucocorticoids in early RA as a disease-modifying antirheumatic drug, in which dosages of 5 to 10 mg prednisone equivalent during 1 to 2 years are used, for prolonged treatment the glucocorticoid dosage should be kept to a minimum, and a glucocorticoid taper should be attempted in the case of remission or low disease activity; the reasons to continue glucocorticoid therapy should be regularly checked. The need for continuing glucocorticoid treatment should be under constant review, and the dose should be titrated against therapeutic response, risk of undertreatment, and development of adverse effects [70].

Optimal choices regarding the use of glucocorticoids in rheumatic diseases are patient specific. The underlying disease, the presence of co-morbidity, the response to initial treatment, and the development of adverse effects should influence treatment decisions. Monitoring for adverse effects should also be tailored to the patient: specific aspects of individual patients may warrant a higher frequency of monitoring or a more comprehensive set of adverse effects to be monitored. Continuously balancing the benefits and risks of glucocorticoid therapy is recommended.

### Use in pregnancy

The fetus is protected from maternal glucocorticoids since glucocorticoids bound to proteins cannot pass the placenta, and the enzyme 11β-hydroxysteroid dehydrogenase in the placenta converts cortisol and prednisolone into the inactive 11-dehydro-prohormones. The prednisolone maternal-to-fetal blood concentration ratio is therefore about 10:1. The risk of adverse effects of antenatal exposure to glucocorticoids such as reduced intrauterine growth and birth weight, neurocognitive adverse effects and oral cleft seems dependent on the dose, preparation, duration of therapy and stage of pregnancy [122]. Avoiding high doses (such as 1 to 2 mg/kg prednisone equivalent) is advised in the first trimester of pregnancy, whereas low to moderate doses of prednisone seem to be safe [122,123].

## Future

New biological therapies have not replaced glucocorticoids, and probably will not replace them in the near future, as anchor drugs in therapeutic strategies for rheumatic diseases. Future research should therefore stay focused on the mechanisms causing beneficial and harmful effects and on predictive factors for the effects of glucocorticoids. Ultimately, response to glucocorticoids should preferably be predictable for the individual patient; studies on genomics and proteomics are being performed currently to this aim.

Apart from prediction of the treatment effects, there is an ongoing quest for new glucocorticoids without potential to cause harmful effects. Optimized glucocorticoids, such as selective glucocorticoid receptor agonists, are being developed to minimize the adverse effects many patients experience, especially if glucocorticoids are given at higher dosages over longer periods of time [124]. The most important approach to optimize the risk-benefit ratio of glucocorticoids is to understand in more detail how the molecular mechanisms of genomic and nongenomic glucocorticoid actions - and their dose dependency - mediate the clinically wanted benefits but also the known adverse effects [125]. During past years, it has become evident that separation of the beneficial effects from the harmful effects is a more complicated process than anticipated [19,23]. Further research on this topic is ongoing [126], but a breakthrough for clinical practice will probably not occur within the next few years.

In some decades to come, we will hopefully be able to prescribe new drugs with glucocorticoid actions with an improved risk-benefit ratio. Moreover, it might be possible to adjust the use of glucocorticoids and other medication to specific individual patients' needs, characteristics and prognostic factors: personalized medicine. This would lead to a more effective inhibition of the inflammatory diseases with less adverse effects. While this has not been achieved, basic research on glucocorticoid effects and detailed reports on therapeutic efficacy and occurrence of adverse effects will be valuable in weighing benefits and risks in clinical practice.

## Conclusion

Glucocorticoids are very effective anti-inflammatory and immunosuppressive drugs, but their use is restrained by fear for and occurrence of adverse effects. Many of the adverse effects can be avoided or dealt with when glucocorticoids are used prudently. Optimal choices regarding the use of glucocorticoids in rheumatic diseases are patient specific, as is monitoring for adverse effects. There is an ongoing quest for new glucocorticoids without the potential to cause harmful effects, such as selective glucocorticoid receptor agonists, but the application of a new compound in clinical practice will probably not occur within the next few years.

## Abbreviations

NSAID: nonsteroidal anti-inflammatory drug; RA: rheumatoid arthritis; SLE: systemic lupus erythematosus.

## Competing interests

The authors declare that they have no competing interests.

## Authors' contributions

The authors participated in the design of and helped to draft the manuscript. All authors read and approved the final manuscript.

## Declaration

This article has been published as part of *Arthritis Research & Therapy* Volume 16 Suppl 2, 2014: At the interface between immunology and endocrinology in rheumatic diseases. The full contents of the supplement are available at http://arthritis-research.com/supplements/16/S2.

This supplement was proposed, developed and commissioned by *Arthritis Research & Therapy *and was funded by an educational grant from Horizon Pharma Inc. All published articles were independently prepared by the authors and have undergone peer review in accordance with the journal's standard policies and processes. Horizon Pharma Inc had no input into the topics covered or the articles themselves. The Supplement Editor was appointed by the journal and declares that they have no competing interests.
